# Educational level and the use of mental health services, psychotropic medication and psychotherapy among adults with a history of physician diagnosed mental disorders

**DOI:** 10.1177/00207640221111091

**Published:** 2022-07-12

**Authors:** Marie Halme, Päivi Rautava, Lauri Sillanmäki, Markku Sumanen, Sakari Suominen, Jussi Vahtera, Pekka Virtanen, Paula Salo

**Affiliations:** 1 Department of Psychology, University of Turku, Finland; 2 Department of Public Health, University of Turku, Finland; 3 Turku Clinical Research Centre, Turku University Hospital, Finland; 4 Department of Public Health, University of Helsinki, Finland; 5 Faculty of Medicine and Health Technology, Tampere University, Finland; 6 School of Health Sciences, University of Skövde, Sweden; 7 Department of Public Health and Centre for Population Health Research, University of Turku, Finland; 8 Faculty of Social Sciences, Tampere University, Finland; 9 Finnish Institute of Occupational Health, Helsinki, Finland

**Keywords:** Educational level, socioeconomic status, mental health service use, psychotropic medication, psychotherapy

## Abstract

**Background:**

The prevalence of mental disorders is increased among people of low socioeconomic status or educational level, but it remains unclear whether their access to treatment matches their increased need.

**Aims:**

Our objective was to examine whether educational level as an indicator of socioeconomic status is associated with use of mental health services, psychotropic medication and psychotherapy in Finland.

**Method:**

Cross-sectional data from a follow-up survey of a longitudinal, population-based cohort study were used to form a sample of 3,053 men and women aged 24 to 68 with a current or previous physician diagnosed mental disorder. The prevalence of mental disorders, mental health service use and educational level were assessed with self-report questionnaire. Educational level was determined by the highest educational attainment and grouped into three levels: high, intermediate and low. The associations between educational level and mental health service -related outcomes were assessed with binary logistic regression. Covariates in the fully adjusted model were age, gender and number of somatic diseases.

**Results:**

Compared to high educational level, low educational level was associated with higher odds of using antidepressants (OR 1.35, 95% CI [1.09, 1.66]), hypnotics (OR 1.33, 95% CI [1.07, 1.66]) and sedatives (OR 2.17, 95% CI [1.69, 2.78]), and lower odds of using mental health services (OR 0.80, 95% CI [0.65, 0.98]). No associations were found between educational level and use of psychotherapy.

**Conclusions:**

The results do not suggest a general socioeconomic status related mismatch. A pharmacological emphasis was observed in the treatment of low educational background participants, whereas overall mental health service use was emphasized among high educational background participants.

## Introduction

Mental disorders are common, with an estimated 38% of Europeans suffering from at least one mental disorder annually (Wittchen et al., 2011). It has been estimated that 35% to 50% of people with serious mental health illnesses did not receive treatment in developed countries during a 12-month period (Demyttenaere et al., 2004). This percentual gap between people in need of mental health care and people who receive such treatment is referred to as the mental health treatment gap (Kohn et al., 2004) and it could also explain some of the debilitating effects mental illnesses have (Alegría et al., 2000). Possible explanations for the unmet needs in use of mental health services include low perceived need as well as attitudinal barriers, such as negative health beliefs, and structural barriers, such as financial barriers and unavailability of services (Prins et al., 2008). Other contributing factors are age and gender, with older adults and men using less mental health services (Karlin et al., 2008; Smith et al., 2013), although this association may also depend on the severity of the disorder and the type of services used (Gagné et al., 2014; Kovess-Masfety et al., 2014; Rhodes et al., 2002).

One possible factor explaining the unmet needs in mental health care is socioeconomic status (SES) that seems to have a bidirectional connection with mental health. SES is commonly operationalized as education, social class, income or a combination of these. In the current study, educational level was used as a proxy indicator of SES as it is also a strong predictor of for example income and employment, making it a commonly used indicator of SES in research (Galobardes et al., 2006). Low SES can be a risk factor for developing a mental health illness or falling more severely ill (Fryers et al., 2003; Kivimäki et al., 2020; Lorant et al., 2003), and having a mental health illness can lead to lower SES through missed days of work and thus, lower educational achievement and income (Barbato et al., 2014). A multi-cohort study, which included also the HeSSup cohort mapped morbidity from electronic health records. The findings showed that low socioeconomic status measured by educational attainment is a risk factor for a spectrum of interconnected diseases and health conditions and highlighted the importance of mental health problems and substance abuse in the cascade of socioeconomically patterned physical illnesses (Kivimäki et al. 2020).

Lower mental health care utilization has also been associated with low educational level (Wang et al., 2007), among other structural barriers related to SES, such as low income (Wang et al., 2007), financial difficulties (Simon et al., 2004), low neighbourhood SES (Steele et al., 2006) and not having health insurance (Walker et al., 2015). Low SES has been associated with lower mental health care utilization due to attitudinal reasons, such as stigma surrounding mental health illnesses (Jagdeo et al., 2009; Saldivia et al., 2004).

Barriers to treatment seem to vary between countries (Sareen et al., 2007; Simon et al., 2004), possibly influenced by cultural differences, income, health care structure and funding (Barbato et al., 2014). In Finland, relatively little information exists about possible associations between socioeconomic factors, such as educational level, and the use of mental health care services. In one Finnish study, level of education or income were not associated with mental health service use among people with depression or anxiety (Hämäläinen et al., 2008). In a large cohort of public sector employees, no overall association between socioeconomic position and antidepressant treatment were observed while, among men, a lower antidepressant use was found to associate with low socioeconomic position. However, both among men and women, employees of low socioeconomic position had an increased risk of mental health related mortality, as indicated by suicides and deaths from alcohol-related causes, as well as all-cause mortality (Kivimäki et al., 2007). In a recent look at the general health service use in Finland people with high income had more annual doctor visits and used more private sector and occupational health services than those in the lowest income groups (Kajantie, 2014). Unemployed and non-permanently employed respondents also used less physician services than permanently employed (Virtanen et al., 2006). These results may suggest that a link between SES indicators and mental health service use could also exist in Finland.

Identifying barriers to mental health treatment is important for reducing disability caused by mental disorders. In this study using data from a Finnish population-based cohort study, the main interest was to examine whether educational level, also understood as a proxy to SES, is associated with the overall use of mental health services, psychotropic medication and psychotherapy. Based on previous findings from international studies and the health care system in Finland, which offers faster access to services through private sector or occupational health care than public sector, we hypothesized that lower educational level would be associated with poorer access to mental health services.

## Methods

### Participants and study design

**Figure 1.**
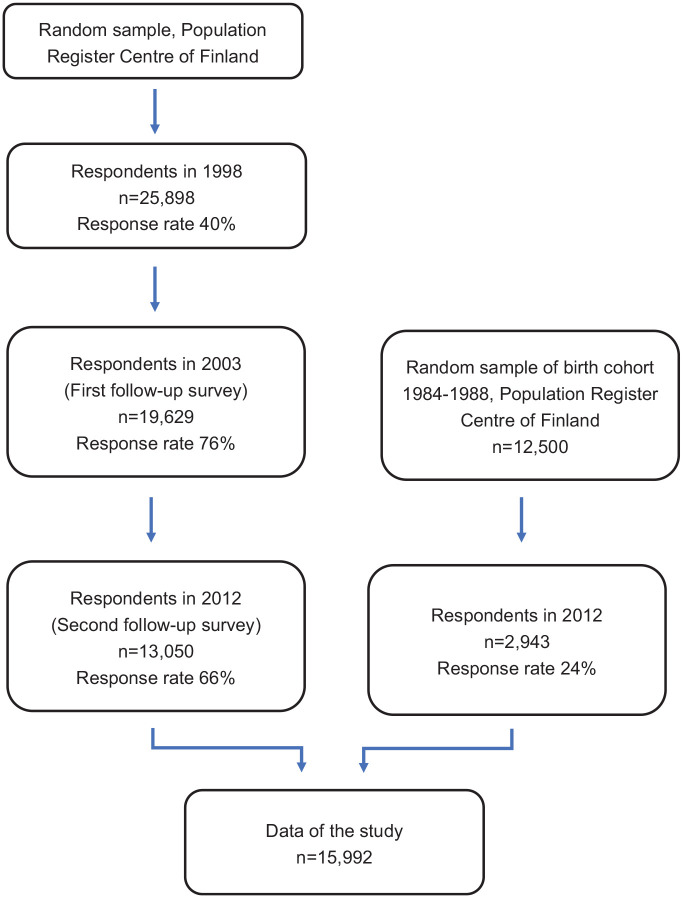
Flow chart of study population.

The data used in this study has been derived from the Health and Social Support (HeSSup) study. HeSSup is a longitudinal Finnish population-based cohort study covering areas of life such as health, stress, lifestyle, personality, relationships, social support, social background and education. The sample was representative at baseline in 1998 of age groups 20 to 24, 30 to 34, 40 to 44 and 50 to 54 years (Korkeila et al., 2001). The questionnaires were sent out in 1998, 2003 and 2012. The first postal survey in 1998 was returned by 25,898 respondents (response rate 40%). The first follow-up survey in 2003 was returned by 19,629 respondents (response rate 76%), who had previously responded to the first postal survey, and the second follow-up survey in 2012 was returned by 13,050 respondents (response rate 66% of those who responded to the 2003 survey). In addition to the respondents from the previous two surveys, the 2012 follow-up survey was sent out to 12,500 randomly selected young adults born in 1984 to 1988 and was returned by 2,942 respondents (response rate 24%). Flow chart of the study population is presented in Figure 1. The HeSSup study has been approved by the Turku University Central Hospital Ethics Committee.

In this study, we used data from the 2012 follow-up study including the original participants and the young adults’ cohort (total *N* = 15,993). The criteria for inclusion were having complete data on all relevant variables and having a mental disorder during the time of survey response or earlier. Information on mental disorders was based on a question ‘Has a doctor ever told you that you have or have had any of the following diseases’. The survey options included both somatic illnesses and mental disorders. Out of diseases listed, we included all the following inquired mental health related options: depression, panic disorder, eating disorder and other mental disorder. After excluding cases that did not fulfil the inclusion criteria, 3,053 participants remained in the sample. In the final sample 73% of the respondents were women and the mean age was 46.1 years (*SD* = 14.1).

### Level of education

Level of education comprised of three levels: high, intermediate and low. High educational level included education at college level or higher. Intermediate educational level included vocational education and post-secondary education. Low educational level included basic education and non-formal vocational educational training, such as vocational courses or vocational apprenticeship training. Three-level grouping was adopted from other studies that have used the same dataset and examined educational level (Heikkilä et al., 2012; Kivimäki et al., 2012).

### Outcomes

This study had five outcomes: use of (1) any mental health services, (2) psychotherapy services, (3) antidepressant medication, (4) sedatives and (5) hypnotics. Mental health service use was measured with a question ‘Have you used the following health services. If yes, when?’ The response options were: ‘never’, ‘during the last year’, ‘during last 1 to 5 years’ and ‘earlier (than during the last 5 years)’. The answer was dichotomized: it was coded as ‘no’, if the participant had selected the option ‘never’ and ‘yes’ in all other cases.

Psychotropic medication use was measured with a question ‘Have you used the following medications or products during the last year?’ Out of the medications and products included in the question, antidepressants, sedatives and hypnotics were considered as psychotropic medications. The response options were: ‘I have not used this medication’, ‘I have used this medication for less than 10 days’, ‘for 10 to 59 days’, ‘for 60 to 180 days’ and ‘for longer than 180 days’. Answers to each psychotropic medication question were dichotomized. If the participant had selected the option ‘never’, the answer was coded as ‘no’. All other answers were coded as ‘yes’.

Psychotherapy service use was inquired with a question about different forms of rehabilitation. In the survey, rehabilitation options were physiotherapy, occupational therapy, vocational rehabilitation, psychotherapy and other form of rehabilitation. Of the list of different rehabilitation forms, only psychotherapy was included. Other rehabilitations included only physical rehabilitation or a combination of physical and mental rehabilitation, like vocational rehabilitation, which can include both physical and psychotherapeutic elements, as well as psychoeducation. The response options were ‘never’, ‘during the years of 2010 to 2011’, ‘during years 2003 to 2009’, ‘during years 1998 to 2002’ and ‘earlier than 1998’. If the participant had selected the option ‘never’, the answer was coded as ‘no’. If the participant had responded positively concerning any period earlier than the year 1998 up to the years 2010 to 2011, the answer was coded as ‘yes’.

### Covariates

Age, gender (male/female) and number of somatic diseases were included as covariates, as they were possible confounding factors. Information on gender and age were obtained from the register of the Population Register Centre and number of somatic diseases were based on survey responses. Information on the number of somatic diseases was based on a question ‘Has a doctor ever told you that you have or have had any of the following diseases’. Out of the diseases listed, 28 diseases were considered somatic and included in the analyses. A new variable indicating the respondent’s total number of somatic diseases was created by summing values of 0 (no disease) and 1 (disease), that were assigned to each response.

### Statistical analyses

The associations between educational level and background information were analysed with Pearson’s χ^2^-test for gender and mental disorders (depression, panic disorder, eating disorder and other mental disorders), and with non-parametric alternative for analysis of variances (Kruskal-Wallis *H* test) for age and number of somatic diseases. Mann-Whitney *U*-test was used to compare differences between the groups. The associations between the independent variable (educational level) and the dependent variables (use of mental health services, antidepressants, sedatives, hypnotics or psychotherapy) were analysed with binary logistic regression models. The dependent variables were dichotomized (service use or no service use), and the independent variable was categorized in three classes (low/intermediate/high) with high educational level as the reference category.

Two logistic regression models were formed. In Model 1, only the associations between educational level and each outcome were analysed. Model 2 was adjusted for age, gender and number of somatic diseases. In the Model 2, interaction effects between educational level and gender and between educational level and age were analysed separately. The statistical analyses were conducted using IBM SPSS Statistics version 26 (IBM Corp, Armonk, NY, USA). For the binary logistic regression analyses, odds ratio (OR) and 95% confidence interval are reported. The explanatory power of the models was estimated with Nagelkerke *R*^2^ and is reported for the adjusted models.

## Results

### Descriptive statistics

Out of the 15,993 participants, 3,053 reported a physician diagnosed depression, panic disorder, eating disorder or other mental disorder. Depression was the most frequently reported mental disorder with 2,379 (77.9%) participants reporting it. Panic disorder was reported by 1,022 (33.5%), eating disorders by 339 (11.1%) and other mental disorders by 604 (19.8%) participants. Most of the participants (68.6%) reported having only one mental disorder, whereas two or more mental disorders were reported by 31.4%. In the final sample, the mean age of participants was 46.1 years, 74.1% were women, 21.0% had no formal educational degree after basic primary education (low level of education), 43.5% had completed vocational education (intermediate level of education) and 35.6% had completed a higher educational degree (high level of education).

Descriptive statistics of the participants are given in Table 1. Panic disorder was more commonly reported in participants with low educational background (χ^2^(2) = 31.71, *p* < .001), whereas eating disorders were more common in those with high education (χ^2^(2) = 26.70, *p* < .001). There were no differences between the educational groups in the reported prevalence of depression or other mental disorders. Women were more likely to have a high educational degree than men, (χ^2^(2) = 28.23, *p* < .001). Participants with high educational background were younger (*U* = 256,747, *p* < .001) and had less somatic diseases (*U* = 262,182, *p* < .001) than participants with low educational background.

**Table 1.** Descriptive statistics.

	Level of education			
	All	Low	Intermediate	High
	*N*	*N* (%)	*N* (%)	*N* (%)
Gender				
Men	791	205 (25.9)	360 (45.5)	226 (28.6)
Women	2,262	435 (19.2)	967 (42.7)	860 (38.0)
Depression				
Yes	2,379	499 (21.0)	1056 (44.4)	824 (34.6)
No	674	141 (20.9)	271 (40.2)	262 (38.9)
Panic disorder				
Yes	1,022	262 (25.6)	475 (46.5)	303 (29.6)
No	2,031	378 (18.6)	870 (42.8)	783 (38.5)
Eating disorder				
Yes	339	52 (15.3)	124 (36.6)	163 (48.1)
No	2,714	588 (21.7)	1 203 (44.3)	923 (34.0)
Other mental disorder				
Yes	604	143 (23.7)	261 (43.2)	200 (33.1)
No	2,449	497 (20.3)	1 066 (43.5)	886 (36.2)
	*M* (*SD*)	*M* (*SD*)	*M* (*SD*)	*M* (*SD*)
Age (years)	46.09 (14.12)	48.91 (15.49)	48.15 (13.37)	41.90 (13.17)
Number of self-reported somatic diseases	3.34 (3.46)	3.75 (3.38)	3.65 (3.40)	2.72 (3.49)

*Note*. Summary of the 2012 follow-up data from the nationwide HeSSup study of Finnish adults.

### Mental health services

Unadjusted, low educational level was associated with 0.64-fold odds of using mental health services (95% CI [0.52, 0.78]) compared to high educational level (Table 2). Intermediate educational level was associated with 0.75-fold odds of using mental health services (95% CI [0.64, 0.88]) compared to high educational level. After adjustments for age, gender and number of somatic diseases, low educational level was associated with 0.80-fold odds of using mental health services (95% CI [0.65, 0.98]). The association between intermediate educational level and the use of mental health services was no longer statistically significant. The explanatory power of the adjusted model was low (Nagelkerke *R*^2^ = .070).

**Table 2.** Associations between educational level, psychotherapy and psychotropic medications in the 2012 follow-up data from the nationwide HeSSup study of Finnish adults.

Educational level	*N*	Percentual amount^a^	Odds of mental health service use, psychotherapy and psychotropic medication use	
			Model 1	Model 2
			OR [95% CI]	OR [95% CI]
			Use of mental health services	
High	1,086	57.7	1.0 (Ref)	1.0 (Ref)
Intermediate	1,327	50.6	0.75 [0.64, 0.88]	0.92 [0.78, 1.09]
Low	640	46.6	0.64 [0.52, 0.78]	0.80 [0.65, 0.98]
			Psychotherapy	
High	1,084	26.9	1.0 (Ref)	1.0 (Ref)
Intermediate	1,327	24.0	0.86 [0.72, 1.03]	0.87 [0.72, 1.05]
Low	640	22.3	0.78 [0.62, 0.98]	0.80 [0.64, 1.01]
			Use of antidepresssants	
High	1,086	31.3	1.0 (Ref)	1.0 (Ref)
Intermediate	1,327	35.5	1.21 [1.02, 1.43]	1.26 [1.05, 1.49]
Low	640	37.0	1.29 [1.05, 1.59]	1.35 [1.09, 1.66]
			Use of hypnotic drugs	
High	1,084	25.5	1.0 (Ref)	1.0 (Ref)
Intermediate	1,327	29.5	1.22 [1.02, 1.47]	1.13 [0.94, 1.36]
Low	640	33.3	1.46 [1.18, 1.80]	1.33 [1.07, 1.66]
			Use of sedatives	
High	1,084	14.8	1.0 (Ref)	1.0 (Ref)
Intermediate	1,327	19.5	1.40 [1.13, 1.74]	1.46 [1.17, 1.82]
Low	640	26.6	2.08 [1.63, 2.65]	2.17 [1.69, 2.78]

*Note*. Model 1: Unadjusted. Model 2: Adjusted for age, gender and number of somatic diseases. OR = odds ratio; CI = confidence interval.

a The percentual amount of people that had used the service in question in each educational group.

In the adjusted model, there were no interactions between educational level and gender (*p* = .900), but there was an interaction between educational level and age (*p* = .019). After analysing these age groups separately, in the second oldest age group, that is, 54- to 58-year-olds, low educational level was associated with 0.54-fold odds of using mental health services (95% CI [0.35, 0.83]) compared to high educational level. Intermediate educational level was associated with 0.63-fold odds of using mental health services (95% CI [0.44, 0.91]). In other age groups, the associations between educational level and mental health service usage were not statistically significant.

### Psychotherapy

In Model 1, low educational level was associated with 0.78-fold odds (95% CI [0.62, 0.98]) of using psychotherapy services and intermediate educational level was associated with 0.89-fold odds (95% CI [0.72, 1.03]) compared to high educational level. After adjusting the model for age, gender and number of somatic diseases the associations between low or intermediate educational level and psychotherapy were no longer statistically significant. There were no statistically significant interactions between educational level and gender (*p* = .493) or age (*p* = .396)

### Psychotropic medication

In the associations between educational level and use of psychotropic medication, intermediate educational level was associated with 1.21-fold odds of antidepressant use (95% CI [1.02, 1.43]), 1.22-fold odds of hypnotic drug use (95% CI [1.02, 1.47]) and 1.40-fold odds of sedative use (95% CI [1.13, 1.74]) compared to high educational level. Low educational level was associated with 1.29-fold odds of antidepressant use (95% CI [1.05, 1.59]), 1.49-fold odds of hypnotic drug use (95% CI [1.18, 1.80]) and 2.08-fold odds of sedative use (95% CI [1.63, 2.65]) compared to high educational level.

In Model 2, intermediate educational level was associated with 1.26-fold odds of antidepressant use (95% CI [0.94, 1.36]) and 1.46-fold odds of using sedatives (95% CI [1.17, 1.82]). The association between intermediate educational level and hypnotic drug use was not statistically significant in the adjusted model. Low educational level was associated with 1.35-fold odds of antidepressant use (95% CI [1.09, 1.66]), 1.33-fold odds of hypnotic drug use (95% CI [1.07, 1.66]) and 2.17-fold odds of sedative use (95% CI [1.69, 2.78]). The explanatory power of the adjusted models was low for all three types of psychotropic medication; Nagelkerke *R*^2^ = .005 for antidepressants, Nagelkerke *R*^2^ = .014 for hypnotics and Nagelkerke *R*^2^ = .024 for sedatives.

In the adjusted model, no interactions were found between gender and educational level in any type of psychotropic medication (*p* = .801 for antidepressants, *p* = .902 for hypnotic drugs and *p* = .767 for sedatives), or between age and educational level for sedatives (*p* = .179). There were, however, interactions between age and educational level for antidepressants (*p* = .089), as well as hypnotic drugs (*p* = .001). After analysing the age groups separately, in the youngest age group, that is, 24- to 28-year-olds, intermediate educational level was associated with 1.65-fold odds of using antidepressants (95% CI [1.11, 2.44]) and with 1.82-fold odds of using hypnotic drugs (95% CI [1.17, 2.83]), compared to high educational level. Low educational level was associated with 2.45-fold odds of using antidepressants (95% CI [1.61, 3.74]) and with 3.62-fold odds of using hypnotic drugs (95% CI [2.00, 5.00]), compared to high educational level. The associations between educational level and psychotropic medication use were not statistically significant in other age groups.

## Discussion

### Main results

The study indicated a potential link between educational level and mental health service use in Finland. Educational level was associated with all mental health service-related outcomes but psychotherapy after controlling for confounding factors. Low educational level was associated with reduced odds of using mental health services, while high educational level was associated with reduced odds of being treated by antidepressants, hypnotics and sedatives. Intermediate educational level was also associated with increased odds of using antidepressant, hypnotics and sedatives, although the association was weaker than in the low education group.

Interactions between age and educational level in mental health service use and psychotropic medication use were observed. After analysing age groups separately, both low and intermediate educational level were associated with reduced odds of using mental health services in the age group of 54- to 58-year-olds, while high educational level was associated with reduced odds of using antidepressants or hypnotic drugs in the age group of 24- to 28-year-olds. No interactions between gender and educational level were observed.

More common use of psychotropic drugs and less common use of mental health services in the lower educational groups as compared to the higher ones were observed. Possible explanations for this could be related to the association between high education and higher income (Tamborini et al., 2015), and between low SES and poor health literacy (Stormacq et al., 2019). It is possible that those with higher educational level could have better access to the more expensive private sector health care, which in Finland is available with considerably shorter waiting lists than public sector services. Higher educational level and thus possibly better health literacy could also lead to an increase of knowledge about different treatment options, and how to access them. However, since there was no difference in the use of psychotherapy, this might not be the case in these data.

The findings regarding the use of mental health services are in line with many international studies that have reported a positive relationship between indicators of SES and mental health service use (Saldivia et al., 2004; Steele et al., 2006; Wang et al., 2007). Nevertheless, there have also been studies who have failed to show such a relationship (Hämäläinen et al., 2008). Some of the variation in results could be explained with differences between countries in health care systems and levels of income and education. However, this does not explain the difference in the results between the current study and an earlier Finnish study by Hämäläinen et al. (2008) in which there were no observed associations between level of education or income and mental health service use.

The differences are likely due to differences between study populations, sample sizes and methodological choices. The Hämäläinen et al. (2008) study used a diagnostic interview (Composite International Diagnostic Interview [CIDI]) to determine the prevalence of major depressive disorder, anxiety disorders, alcohol dependency and dysthymia for the past 12 months. This diagnostic interview provides a more reliable timing of mental disorders and mental health service use than the self-report method of the current study, which had no specified time span for the occurrence of mental disorders and service. It should also be considered that the current study had a larger sample than the study by Hämäläinen et al. (2008; 3,053 vs. 540), which made it possible to detect smaller effects.

The finding that psychotropic medication was more commonly used in the youngest age group is somewhat inconsistent with results from other studies. The typical finding is that psychotropic medication use tends to associate with older age (Alonso et al., 2004; Beck et al., 2005), which can be explained for example with the increasing incidence of depression with age (Kessler et al., 2005). Explanations for these deviating results could include cultural differences and a possible increase in the incidence of psychotropic medication use among young adults. Another Finnish study found that the number of children and young adults using psychotropic medications tripled between years 1996 and 2007 (Autti-Rämö et al., 2009). Although the exact mechanisms underlying the current study’s finding of greater odds for use of psychotropic medication among young adults are unclear, the finding remains interesting.

Overall, mental health service use tends to be less common in older age groups, which has been attributed to for example lower perceived need for mental health care among older adults (Karlin et al., 2008). A similar effect was found in the present study in the second oldest age group but not in the oldest one. It could be possible that in the oldest age group of 64- to 68-year-olds overall health care utilization increases, which could reflect to greater mental health care utilization for those in need of it.

Although the associations between the mental health-related outcomes and educational level were statistically significant, the explanatory power of all adjusted models was low. The Nagelkerke *R*^2^-values ranged from .005 (psychotherapy and antidepressants) to .070 (mental health service use). Values below .12 are considered low, thus the explanatory power of the adjusted models was very low, which must be taken in consideration when interpreting the present results. However, since many factors contribute to the use of mental health services, it could be expected that solely one factor, in this case educational level, could not show a very high inherent explanatory power.

### Strengths and limitations

A major strength in the current study was that the original HeSSup study was a large population-based study, making the original sample representative of the Finnish population. Though the participation rate in the HeSSup study was rather modest (40.0% in the initial postal survey), according to a drop-out analysis, the respondents from the first survey can still be considered representative of the concurrent general population, particularly in relation to morbidity (Korkeila et al., 2001). Moreover, it is unlikely that a modest response rate in itself would have biased the associations now studied. The non-response of the original sample was further explored by Suominen et al. (2012) in a register-based mortality analysis of respondents and non-respondents, which revealed only slight differences according to response status. The sample of the current study was derived from the original sample by only including participants with self-reported mental disorders.

The current study also carries limitations. First, the data used were cross-sectional and thus, causal connections between educational level and mental disorders cannot be established. Although the possible effects of educational level on mental disorders were examined, it is possible, and to some extent likely, that the connections between these variables are bidirectional. It is also possible that suffering from a mental disorder may have preceded the completion of education, especially in the young adults’ cohort. Second, there were no data available about the exact time the participant had used mental health services or experienced the reported disorders. The results might have been influenced by how much time had passed between use of service and participation in the study, as well as differences in how the respondents define mental health services and service use. Thus, it cannot be determined if the use of mental health services coincided with the mental disorder or the treatment of it, although this seems probable. Several earlier periods with disturbing mental health problems might have occurred accompanied by varying help seeking behaviour.

The method of data collection by a postal survey also carries some limitations. In a self-report questionnaire, it is possible that some participants’ interpretation of a question could influence their answer. Also, the history of mental disorders was inquired by presenting a list of different somatic and mental illnesses and disorders, and thus, all mental disorders might not necessarily have been reported. Hence, it is possible, that the inclusion criteria might have influenced the current study sample.

### Suggestions for future research and implications

The results from this study suggested no general socioeconomic status related mismatch, but a pharmacological emphasis was observed in the treatment of low educational background participants, whereas overall mental health service use was emphasized among high educational background participants. In future research the influence of different indicators of SES on the use of mental health services, as well as their possible causal connections should be studied further. Moreover, research is also needed on other factors possibly contributing to socioeconomic disparities in mental health service use. Based on results from previous studies, such factors could include differences in attitudes, perceived stigma and costs and availability of mental health services (Jagdeo et al., 2009; Walker et al., 2015), as well as severity of the disorder, which may be associated with how different barriers to mental health care are perceived (Andrade et al., 2014; Mojtabai et al., 2011). Using diagnostic interviews or data from national health registers to verify diagnoses and service use, more information about possible differences of mental health service use between different types of disorders in combination with varying degree of severity could be obtained. Such information could be useful for future development of mental health services and could provide important information on the mental health treatment gap to help policy makers to target actions more efficiently.
